# Bio-Based Pectin-Calcium Film and Foam Adsorbents with Immobilized Fe–BTC MOF for Water Contaminant Removal

**DOI:** 10.3390/polym18020171

**Published:** 2026-01-08

**Authors:** Francesco Coin, Carolina Iacovone, Silvina Cerveny

**Affiliations:** 1 Centro de Física de Materiales (CSIC, UPV/EHU)—Materials Physics Center (MPC), Paseo Manuel de Lardizabal 5, 20018 San Sebastián, Spain; ciacovone001@ikasle.ehu.eus; 2 Donostia International Physics Center (DIPC), Paseo Manuel de Lardizabal 4, 20018 San Sebastián, Spain

**Keywords:** bio-based adsorbents, metal-organic framework (MOF), water purification, pectin-calcium films and foams, iron-based-MOF, adsorption

## Abstract

Metal-organic frameworks (MOFs) offer high porosity for water remediation but face challenges in handling as powders. We address these limitations by physically immobilizing Fe–BTC MOF within calcium-crosslinked low-methoxyl pectin matrices (PE–Ca–MOF). Solvent-cast films and freeze-dried foams were fabricated using water-based and polyvinylpyrrolidone (PVP)-assisted Fe–BTC dispersions, preserving MOF and pectin structures confirmed by FT–IR. PVP improved Fe–BTC dispersion and reduced particle size, enhancing distribution and plasticizing the matrix proved by DSC. Incorporation of water-dispersed Fe–BTC increased the equilibrium adsorption capacity but reduced the initial adsorption rate, while the PVP-assisted foam further enhanced uptake in comparative batch tests through its more open porous structure. At pH 7, PE–Ca–5%MOF films showed high adsorption capacities and removal efficiencies for paraquat (35.5 mg/g, 70.6%) and tetracycline (14.5 mg/g, 46.8%), while maintaining Zn^2+^ uptake compared to calcium-pectin films without MOF. Adsorption followed pseudo-first-order kinetics and Langmuir isotherms. Green regeneration with acetic acid enabled >80% capacity retention over five adsorption–desorption cycles. Foam architectures increased porosity and active-site accessibility (SEM), improving performance even at lower MOF loadings. Overall, controlling MOF dispersion and composite morphology enables efficient, reusable, and environmentally friendly bio-based adsorbents for water purification.

## 1. Introduction

Nanotechnology has become a key tool for the next generation of water purification technologies. Various nanomaterials, such as nanofibers [1,2,3], nanoparticles [4], nanotubes [5], biochar [6], and/or mineral clays [7], have been explored for removing pollutants from water. Nanoparticles (NPs) are particularly effective due to their large surface area and high reactivity, enabling efficient pollutant capture [4]. Within the family of NPs, metal-organic frameworks (MOFs) have gained particular attention because of their adjustable porosity and versatile functionality, finding uses in fields such as catalysis, energy storage, drug delivery, gas separation, and, more recently, they have become highly promising adsorbents for environmental water cleanup [8].

In recent years, various MOF materials have been tested for water cleaning and pollutant removal. Examples include: Zr-based UiO-66 and its derivatives for antibiotics/dyes; ZIF-type frameworks (ZIF-8/ZIF-67) for dyes, antibiotics and pesticides; Zr-MOF-808 for PFAS; Fe/Cr MIL-101 and MIL-53 families for dyes and oxyanions; and Ti-based MIL-125 for photocatalytic degradation of organics under UV/visible [9,10,11,12].

MOFs are crystalline porous materials composed of metal ions connected by organic linkers, which create a network with an extremely high porosity. Their ultra-high surface area and tunable surface chemistry enable the capture of a wide range of contaminants from aqueous systems [13]. However, in powder form, MOFs present several practical limitations for direct application in water treatment. Handling fine powder is challenging and can lead to fouling and secondary contamination of treated water, especially at high MOF concentrations. Additionally, if MOFs are not sufficiently water-stable, they may partially dissolve, releasing metal ions and organic linkers into the environment. These limitations have motivated researchers to immobilize MOFs within polymer matrices for improved handling and stability [12].

Despite growing interest in MOF–polymer composites for water treatment [13], three critical gaps limit their practical application and environmental sustainability. First, most studies employ synthetic, non-biodegradable polymers [14] that contradict green chemistry principles and contribute to plastic pollution. Second, the effect of MOF dispersion quality on composite performance is rarely investigated systematically; most studies use a single dispersion method without optimization or comparison, potentially leaving significant performance improvements unexplored. Third, the influence of composite morphology, particularly the comparison between two-dimensional films and three-dimensional porous structures (2D films vs. 3D foams), on adsorption kinetics and capacity remains poorly understood for bio-based matrices. Furthermore, while Fe–BTC (also known as Basolite F300) has demonstrated excellent adsorption capacity for various contaminants in powder form [15,16,17,18,19,20], its integration into sustainable bio-based matrices for multi-contaminant water purification has not been systematically explored.

Pectin, a polysaccharide present in plant cell walls, offers unique advantages and it is particularly promising: (1) abundant availability from citrus and apple processing waste, enabling waste valorization [21]; (2) excellent film-forming properties [22]; (3) tunable ionic crosslinking with Ca^2+^ ions, creating hydrogels with maintained macroscopic integrity [23]; (4) inherent adsorption capacity through carboxyl groups [24,25,26]; and (5) biodegradability, ensuring minimal environmental impact at end-of-life [27]. Moreover, pectin has recently attracted growing interest also for fundamental studies of water mobility confined within nanochannels, which drive the mass transport to active adsorption sites in aqueous media [28,29]. Pectin has many hydroxyl and carboxyl functional groups, which can bind metal ions [23,30,31]. In particular, low-methoxyl pectin (LMP) can be cross-linked (for instance with Ca^2+^ ions) to form insoluble hydrogels composed of “egg-box” dimers, which are the active sites for binding metal ions [31]. Thus, calcium-crosslinked low-methoxyl pectin (PE–Ca) can provide a versatile platform for hosting MOF particles, thereby improving handling and preventing secondary contamination

Therefore, this study aims to develop sustainable PE–Ca–MOF composites using two distinct morphologies (solvent-cast films (2D) and freeze-dried foams (3D)) to investigate structure–performance relationships. We have systematically compared water-based and PVP-assisted MOF dispersion methods and their impact on particle size, distribution, and adsorption performance. We selected Fe–BTC for its availability, low cost, water stability [32], low toxicity [33], and strong affinity for target pharmaceuticals; Fe^3+^ sites and high internal surface area facilitate robust interactions with these molecules [15]. In addition, we have evaluated adsorption performance against three contaminant classes representing diverse physicochemical properties: heavy metals (Zn^2+^), pesticides (paraquat), and antibiotics (tetracycline) and also assessed the adsorption properties of other pharmaceuticals (Atenolol, Metformin, and Metoprolol). Moreover, we have assessed practical reusability and regeneration potential using bio-based acetic acid over multiple adsorption-desorption cycles. To elucidate structure–performance relationships through comprehensive characterization (DLS, FTIR, XRD, SEM, DSC) and adsorption modeling (kinetics and isotherms). This work bridges the gap between fundamental materials science and practical environmental applications, offering a sustainable alternative to synthetic polymer–MOF composites while providing mechanistic insights into dispersion.

## 2. Materials and Methods

### 2.1. Materials

Citrus pectin powder (PE) with nominal values of 91% of galacturonic acid (Gal) and methyl esterification degree (DM) of 9.9% was supplied by Herbstreith & Fox (Neuenbürg, Germany). Sodium chloride (NaCl, product number S9625), Calcium chloride (CaCl_2_, product number C1016), Zinc chloride (ZnCl_2_, product number 208086), Sodium hydroxide (NaOH, product number S5881), Atenolol (ATE, product number A7655), Metformin (MFT, product number 317240), Metoprolol (MET, product number PHR1076), Paraquat (PQ, product number 856177) and Tetracycline (TC, product number 87128) were purchased from Sigma Aldrich (Madrid, Spain). Nitric acid (HNO_3_ 65% *w*/*w*, product number 7697-37-2) was purchased from Scharlab (Barcelona, Spain). NaOH and HNO_3_ were used to adjust the pH. Acetic acid (AA, glacial (99%)) was purchased from Fischer Chemical (Madrid, Spain) (product number UN2789) and used for the desorption experiments. Milli–Q water was used to prepare the solutions.

#### Contaminant Selection Rationale

Three model contaminants were selected to represent diverse physicochemical properties and environmental relevance: (1) Zinc (II) (Zn^2+^) as a representative heavy metal commonly found in industrial effluents from electroplating, mining, and metallurgy [34], with ionic radius of 0.74 Å and +2 charge enabling ion exchange and coordination interactions; (2) Paraquat (1,1′–dimethyl–4,4′–bipyridinium dichloride, MW = 257.16 g/mol) as a widely used herbicide with high aquatic toxicity [35], existing as a di-cation (pKa >> 14) at neutral pH, enabling electrostatic interactions; and (3) Tetracycline (C_22_H_24_N_2_O_8_, MW = 444.43 g/mol) as a broad-spectrum antibiotic frequently detected in wastewater [36], existing as a zwitterion at pH 7 (pKa values: 3.3, 7.7, 9.5) with multiple functional groups enabling complexation, hydrogen bonding, and π–π interactions. This selection enables assessment of adsorbent versatility across different binding mechanisms and molecular sizes. In addition, also three more pharmaceuticals were tested: atenolol, metformin, and metoprolol.

### 2.2. MOF Dispersion Methods

Two dispersion methods were compared to optimize MOF particle size and distribution. In the water-based dispersed method, 1 wt%, 5 wt%, and 10 wt% of Fe–BTC (relative to the amount of pectin) were added to Milli-Q water (50 mL). In PVP-assisted dispersion method, 1% Fe–BTC was dispersed in 50 mL of a 2.5% (*w*/*v*) polyvinylpyrrolidone (PVP) aqueous solution, which served as a surfactant. In both methods, the solutions were continuously stirring and subjected to tip sonication for 30 min (10 s pulses at 35% amplitude) at 70 °C. The quality of dispersion was evaluated using dynamic light scattering (DLS), which revealed reduced particle aggregation in the PVP-assisted dispersion method (see Section 2.3).

### 2.3. Composite Preparation

#### 2.3.1. Film Fabrication (Solvent Casting)

Pectin (PE) was dissolved in deionized water (3 g in 50 mL) under magnetic stirring at 70 °C for 1 h. The solution was then sonicated in a thermal bath at 70 °C for an additional hour to ensure complete PE dissolution.

The solution was kept at the same temperature and the MOF dispersion (water-based or PVP-assisted) was added dropwise under continuous stirring at 300 rpm and constant tip sonication for 30 min (10 s pulses at 35% amplitude) at 70 °C to achieve final MOF loadings of 1 wt%, 5 wt%, and 10 wt% relative to pectin dry weight.

The mixture was cast into Teflon Petri dishes (10 cm diameter) and dried at 23 °C and 70% relative humidity for 24 h and subsequently dried in a vacuum oven at 40 °C for 1 h. The films were immersed in a 500 ppm Ca^2+^ solution (1.25 g/L relative to PE weight) under constant shaking at 125 rpm for 40 min at room temperature to induce ionic crosslinking (external) [37]. To remove excess ions and unbound components, after crosslinking, the materials were washed five times with fresh deionized water (dose 1.25 g/L). The resulting films had a thickness of 0.10 ± 0.01 mm. Films were then dried in a vacuum oven at 40 °C for 1 h and then stored in sealed containers with silica gel desiccant until use.

Consequently, insoluble Ca^2+^-crosslinked PE films with varying MOF concentrations were obtained and labeled as follows: PE–Ca (no MOF), PE–Ca–1%MOF, PE–Ca–5%MOF, PE–Ca–10%MOF (water-dispersion method), and PE–Ca–1%MOF–PVP (PVP-assisted dispersion method).

#### 2.3.2. Foam Fabrication (Freeze-Drying)

Foam samples were prepared following the same protocol as films up to the casting step. The entire solution (50 mL) was poured into flat crystallization dish (15 cm diameter × 7 cm height) covering the entire surface, frozen with liquid nitrogen for 1 h to ensure complete ice crystal formation and then freeze-dried (Schneider Electric, Rueil-Malmaison, France) at −55 °C and <0.1 mbar for 24 h. The sublimation of ice crystals created an interconnected porous network. Foams were also crosslinked, dried and storage as previously explained in Section 2.3.1. As a result, an insoluble, Ca^2+^-crosslinked PE foam containing 1 wt% of MOF (dispersed with PVP-assisted method) was obtained.

#### 2.3.3. Samples Conditioning

All the fabricated materials were dried and stored under vacuum (10^−2^ mbar) at 40 °C for one week and carefully weighed before all structural and adsorption experiments.

### 2.4. Adsorption Experiments

All adsorption experiments were conducted at neutral pH (pH 7) using a temperature-controlled orbital shaker (23 ± 2 °C, 125 rpm, Ovan, Barcelona, Spain). Working at pH 7 is important because it is close to most natural water conditions, minimizing extreme acid/base effects and allowing us to evaluate the material’s performance under realistic conditions. All conclusions about the mechanism discussed in this work refer exclusively to adsorption experiments carried out at neutral pH (pH 7); the pH-dependent behavior of the studied contaminants on these composites remains unknown on the basis of the present data and should be systematically investigated in future studies.

To minimize surface effects and maintain uniform experimental conditions, composite samples were cut into uniform pieces: 22 ± 1 mm in diameter and 0.10 ± 0.01 mm thick for films (approximately 5 mg) and cylinders of 10 ± 1 mm in diameter and 1.00 ± 0.01 mm thick (approximately 5 mg). Contaminant solutions were prepared fresh daily in deionized water and pH-adjusted to 7.0 ± 0.1 using 0.1 M HNO_3_ or NaOH. The composite dose was maintained at 1 g/L (adsorbent dry weight per liter of solution) for all experiments.

Adsorption data are reported as (mean value ± standard deviation), based on experiments carried out in triplicate. Model selection was based on R2 and reduced χ^2^ values, the F-test, the Akaike (AIC), and Bayesian (BIC) information criteria (see Section S1 in the Supporting Material (SM) for details) [38,39,40]. Statistical differences were assessed using a one-way ANOVA on ranks with a 99% confidence level (*p* < 0.01).

#### 2.4.1. Kinetic Experiments

Kinetic experiments were performed at an initial contaminant concentration of 10 mg/L (50 mg/L for Zn^2+^), at pH 7 and at predetermined time intervals (5, 10, 15, 30, 60, 120, 240, 480, 960, and 1440 min).

The adsorption capacity at time t (q_t_, mg/g) was calculated using:

(1) $$q_{t}=\frac{{C_{0}\;-\;C}_{t}}{d}$$

where *C*_0_ is the initial concentration (mg/L), *C_t_* is the concentration at time t (mg/L), *d* is the adsorbent dose (g/L).

#### 2.4.2. Isotherms Experiments

Equilibrium isotherm experiments were conducted by varying the initial contaminant concentration (5, 10, 25, 50, and 75 mg/L) and a fixed contact time of 24 h. The equilibrium adsorption capacity (q_e_, mg/g) was calculated using:

(2) $$q_{e\;}=\frac{{C_{0}\;-\;C}_{eq\;}}{d}$$

where *C*_0_ and *C_eq_* denote the initial and equilibrium concentrations (mg/L) of the crosslinking agent or pollutant, respectively.

The removal efficiency (R_Eff_, %) was determined using the following expression:

(3) $$R_{Eff}(\%)=\;\left(\frac{C_{0}\;-\;C_{eq}}{C_{0}}\right)\times \;100\%$$

#### 2.4.3. Reusability Studies

Reusability was assessed over five consecutive adsorption–desorption cycles using paraquat and tetracycline as contaminant models. After each adsorption cycle (at pH 7, for 1 h), the adsorbent was recovered using 2% *v*/*v* acetic acid solution (at pH 2.3, for 1 h) to desorb the contaminant. Acetic acid was selected as a green regeneration agent due to its bio-based origin (fermentation), low cost, biodegradability, and ability to protonate carboxyl groups without damaging the pectin matrix or MOF structure [41]. Between cycles, samples were washed with water and dried to remove residual reagents. The desorption efficiency (R_des_, %) was calculated as:

(4) $$R_{des}(\%)=\frac{C_{Des}(n)}{{C_{0}\;-\;C}_{eq}(n)}\times \;100\%$$

For each cycle *n*, the initial concentration was *C*_0_ (from the stock solution), the equilibrium concentration after adsorption was *C_eq_*(*n*) (from the treated solution), and the desorption concentration was *C_Des_*(*n*) (from the eluate). After desorption, the adsorbent was washed three times with deionized water, dried to remove residual reagents, and reused for the next cycle.

The retention rate was calculated to assess the stability and reusability of the materials, representing the percentage of adsorption capacity retained in each subsequent cycle compared to the first cycle. The retention rate compares the adsorption capacity in cycle (*n*) to that of the first cycle:

(5) $${Retention\;rate}_{n}(\%)=\frac{q_{n}}{q_{1}}\times \;100\%$$

A high retention rate implies good structural stability and regeneration potential of the adsorbent [42].

#### 2.4.4. Models

The adsorption kinetics were analyzed by fitting the data to empirical kinetic models, namely the pseudo-first-order (PFO) and pseudo-second-order (PSO) equations:

(6) $$\mathrm{PFO}:\;q_{t}=q_{e}(1-e^{-k_{1}t})$$

(7) $$\mathrm{PSO}:\;q_{t}=\frac{q_{e}^{2}k_{2}t}{1+q_{e}k_{2}t}$$

q_e_ is the equilibrium adsorption capacity, k_1_ and *k*_2_ (min^−1^) represent the characteristic time constants of the PFO and PSO models and describe the initial rate of the adsorption process [43].

The adsorption isotherm data were analyzed using the Langmuir (Equation (8)), Freundlich (Equation (9)) models [44]:

(8) $$\mathrm{Langmuir}:\;q_{e}(C_{eq})=\frac{{q_{M}K}_{L}C_{eq}}{1+K_{L}C_{eq}}$$

(9) $$\mathrm{Freundlich}:\;q_{e}(C_{eq})=K_{F}C_{e}^{1/n}$$

In these models, q_e_ is the equilibrium adsorption capacity; q_M_ (mg/g) is the maximum adsorption capacity; K_L_ (L/mg), *n* and K_F_ (L^1/n^mg^1−1/n^g^−1^) are the Langmuir and Freundlich constants, respectively.

The exchange ratio helps to understand the ion exchange mechanism of the adsorbent material for specific ions [45]. The exchange ratio is defined as:

(10) $$Exchange\;ratio=\frac{Moles\;of\;adsorbed\;ion}{Moles\;of\;desorbed\;ion}$$

If the exchange ratio is equal to 1, it indicates a 1:1 molar exchange between the two ions. If the exchange ratio is greater than 1, more of the adsorbed ion is taken up relative to the desorbed ion. However, If the exchange ratio is less than 1, less of the adsorbed ion is taken up relative to the desorbed ion.

### 2.5. Characterization Techniques

The particle size of the dispersed Fe–BTC solutions was measured at 25 °C using a Zetasizer Nano ZS90 (Malvern Instruments Ltd., Worcestershire, UK). Measurements were performed in disposable quartz cuvettes using a backscatter detection angle of 173°. Reported values represent the average of three independent measurements.

The morphology and elemental composition of the pectin-based adsorbents were characterized by scanning electron microscopy (SEM; Quanta 250 ESEM, FEI, Eindhoven, The Netherlands) coupled with energy-dispersive X-ray (EDX) spectroscopy (silicon drift detector, EDAX, Tilburg, The Netherlands). The samples were fixed onto aluminum stubs using double-sided carbon tape, and the chamber pressure was adjusted to 80 Pa in low-vacuum mode. Imaging was performed at an accelerating voltage of 10 kV and a beam current of 5 Pa, using a large-field detector to collect the electrons. Thermogravimetric analysis by TA Instruments (Q500) (New Castle, DE, USA) was employed to assess the water evaporation and thermal stability of PE–Ca samples (5 × 5 × 0.05 mm^3^). Measurements were performed under continuous nitrogen flow of 25 mm^3^/min. To prevent dehydration, the samples were placed on the pan as quickly as possible. The weight reduction was monitored as the temperature increased by 10 K/min for dried PE samples.

Differential scanning calorimetry (DSC) was carried out using a TA Instruments Q2000 equipped with liquid nitrogen cooling. Measurements were performed between 100 and 350 K, under a dry helium purge at 25 mL/min. Samples of approximately 10–15 mg were sealed in hermetic aluminum pans to limit water loss. For annealing experiments, samples were first rapidly cooled at ~20 K/min, then heated at the same rate. Once they reached 115 K, they were further heated to 288 K (annealing temperature) for 4 h. The samples were cooled back to 115 K and heated again to 370 K for a final heating scan. Comparative measurements before and after aging confirmed that no permanent changes occurred due to the annealing procedure.

X-ray powder diffraction (XRD) measurements were carried out on a Philips X’pert PRO automatic diffractometer (Philips, Almelo, The Netherlands) using Cu–Kα radiation (λ = 1.5418 Å) and a θ–θ configuration with a secondary monochromator. The instrument operated at 40 kV and 40 mA using a PIXcel solid-state detector (Philips, Almelo, The Netherlands) that provides an active length of 3.347° in 2θ. A variable divergence slit was employed to maintain a constant illuminated area of 10 mm on the sample surface.

Infrared spectra were collected in ATR mode using a Jasco FT–IR 6300 spectrometer (Jasco Global, Tokyo, Japan) over the range 4000–650 cm^−1^, with a resolution of 4 cm^−1^ and 200 scans per spectrum. After baseline correction, the spectra were normalized to the area of the band at ~1140 cm^−1^, assigned to the glycosidic (–C–O–C) vibration of the polygalacturonic acid backbone (see Section S2 in SM).

A Micromeritics ASAP 2420 instrument (Micromeritics Instrument Corporation, Norcross, GA, USA) was used to record the adsorption–desorption isotherms of nitrogen at −196 °C. Prior to nitrogen adsorption measurements, the samples were degassed under nitrogen at 200 °C for 1 h, reaching a pressure below 10^−4^ mbar The specific surface area was calculated using the Brunauer–Emmett–Teller (BET) equation in the relative pressure range p/p_0_ of 0.05–0.20. Micropore volume (V_MI_) was obtained from a DFT analysis assuming cylindrical pores. For comparison, N_2_ isotherms at 77 K (see Section S3 in SM) for Fe–BTC indicate a pore diameter of 2.0 nm and a BET surface area of 1366 m^2^/g. N_2_ adsorption–desorption isotherms and BET surface area analysis were performed only on the Fe–BTC powder. No BET experiments were conducted on the pectin-based films and foams because the limited surface area would require milling of large sample volumes to reach the minimum mass required for reliable measurements, and the degassing temperatures used for N_2_ sorption are not compatible with the thermal stability of the pectin matrix.

Equilibrium concentrations of calcium and zinc ions were quantified by inductively coupled plasma-atomic emission spectrometry (ICP-AES; Agilent 5100, Agilent Technologies, Santa Clara, CA, USA). The concentrations of the pharmaceutical compounds were determined by ultraviolet–visible (UV–Vis) spectroscopy (Agilent 8453A), using the absorption maxima at 260 nm for PQ and 356 nm for TC.

## 3. Results and Discussion

### 3.1. Characterization of MOF Dispersion

The particle size distribution of Fe–BTC dispersions (1 wt%) was characterized by dynamic light scattering (DLS) to evaluate the effectiveness of the two dispersion methods. Water-based dispersion yielded an average hydrodynamic diameter (Z-average) of (1171 ± 87) nm with a polydispersity index (PDI) of (0.52 ± 0.46), indicating substantial aggregation of MOF particles and moderate size distribution breadth. In contrast, PVP-assisted dispersion significantly reduced the average particle size to (793 ± 36) nm (32% reduction), demonstrating the effectiveness of PVP as a dispersing agent.

The particle size reduction with PVP can be attributed to steric stabilization mechanisms [46]. PVP macromolecules adsorb onto Fe–BTC particle surfaces through coordination between carbonyl groups of the pyrrolidone ring and exposed Fe^3+^ sites on the MOF surface [46]. The adsorbed PVP chains extend into the aqueous medium, creating a steric barrier that prevents particle–particle contact and subsequent aggregation through van der Waals forces [46]. The increased PDI in PVP-assisted dispersion (0.767 ± 0.139) suggests a broader distribution of particle sizes, likely due to variations in PVP adsorption density on different MOF crystal facets.

Despite the high PDI values (>0.4), which indicate polydisperse systems typical of MOF dispersions without extensive surface modification [47], the PVP-assisted method achieved significantly better dispersion quality.

### 3.2. Structural Characterization of PE–Ca–MOF Films Using the Water-Dispersed Method

Initially, the calcium content of all composites after crosslinking was determined to ensure the consistent formation of the “egg–box” structure [37] in pectin. It is important to note that, after crosslinking, the samples were washed five times to remove any excess calcium. Table 1 indicates that the Ca^2+^ content is similar across all samples (PE–Ca and PE with different MOF content (PE–MOF). Since all composites were prepared with the same amount of galacturonic acid (5.86 mmol), the resulting stoichiometric ratio, R = [Ca^2+^]/[GalA], is similar for all composites and meets the requirements of the “egg-box” structure formation [48]. Therefore, the addition of MOF does not interfere with the calcium crosslinking process, in agreement with the finding that pure MOF powder does not adsorb Ca^2+^ ions. Possible release of Fe–BTC particles was evaluated by ICP analysis of the crosslinking solutions and rinsing steps; in all cases the Fe^3+^ concentration was below the instrumental detection limit, indicating that no measurable amount of MOF was released to the liquid phase under the conditions employed.

**Figure 1:**
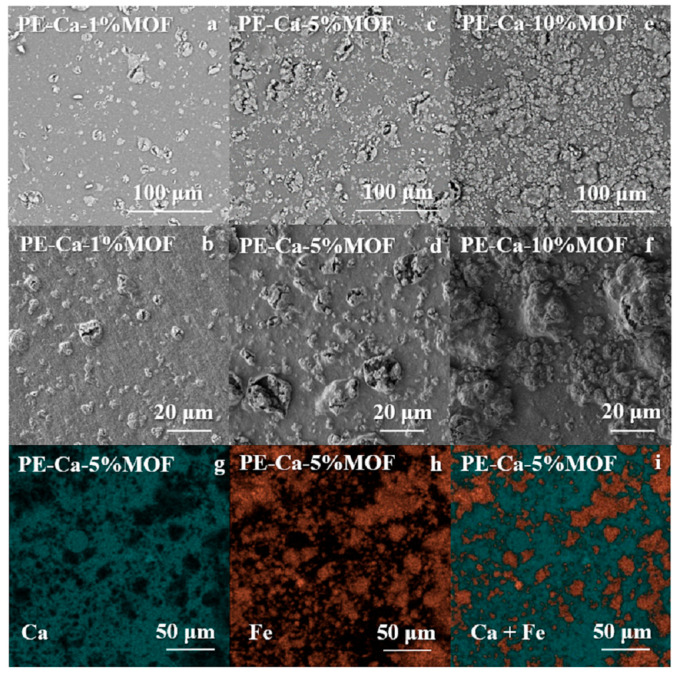
SEM images of (**a**,**b**) PE–Ca–1%MOF, (**c**,**d**) PE–Ca–5%MOF, (**e**,**f**) PE–Ca–10%MOF, and (**g**–**i**) EDAX mapping of PE–Ca–5%MOF.

Figure 1 shows the SEM images of PE–Ca–MOF composites with 1 wt% (a,b), 5 wt% (c,d), and 10 wt% (e,f) of water-dispersed MOF after washing cycles, captured at two magnifications. In the composites, increasing the MOF content results in the formation of larger MOF aggregates within the pectin matrix. EDX elemental mapping for PE–Ca–5%MOF (g–i) shows a uniform distribution of calcium (from the crosslinked matrix) and iron (from the MOF) across the composite surface.

**Figure 2:**
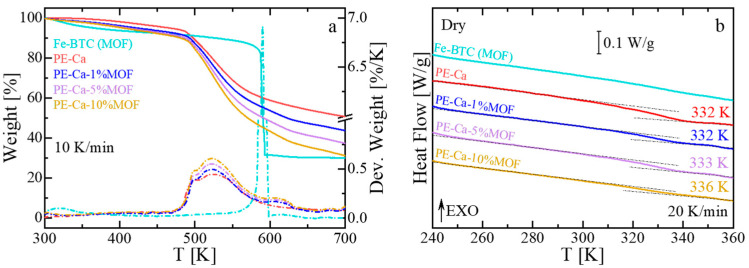
(**a**) Thermogravimetric analysis (TGA, solid line) and derivative weight loss (Deriv. weight, dashed lines) curves of PE–Ca, neat MOF (Fe–BTC), and PE–Ca–MOF water-dispersed composites, recorded at a heating rate of 10 K/min. (**b**) Heat flow as a function of temperature measured by differential scanning calorimetry (DSC) at a heating rate of 20 K/min for dried PE–Ca and PE–Ca–MOF composites after aging. A step change in heat flow indicates the glass transition temperature (T_g_). Numbers on each curve indicated T_g_, determined as the inflection point. The arrow EXO means that any exothermic event (releasing heat) is shown as an upward peak or curve.

TGA of PE–Ca and pure MOF (Fe–BTC) reveals distinct degradation patterns (Figure 2a). PE–Ca has four thermal degradation stages [31]: an initial mass loss due to water evaporation below ~400 K, followed by the release of volatile species between 400 and 475 K [24], the decarboxylation of free and calcium-bound carboxyl groups at 475–575 K, and a carbonaceous residue at higher temperatures [49]. In contrast, Fe–BTC exhibits three main events: water desorption (300–400 K), release of pending and unbounded small molecules (400–500 K), and framework collapse (550–600 K). When incorporated into pectin, Fe–BTC shifts to higher temperatures, with a new broad peak appearing around 615 K. This suggests that the pectin matrix stabilizes the MOF structure, thereby improving its thermal resistance. Figure 2b shows the heat flow versus temperature during the heating run at 20 K/min for Fe–BTC, PE–Ca, and pectin composites at different MOF content. As previously discussed, (see Section 2.5), to detect the glass transition of pectin, it is necessary to perform aging experiments (4 h at 288 K) [31]. For Fe–BTC, no glass transition is observed within the measured range. In contrast, the glass transition temperature of the composites grows from 332 K to 336 K with increasing MOF content, indicating increased rigidity and reduced polymer chain mobility, consistent with the observed rise in brittleness at higher MOF contents.

**Figure 3:**
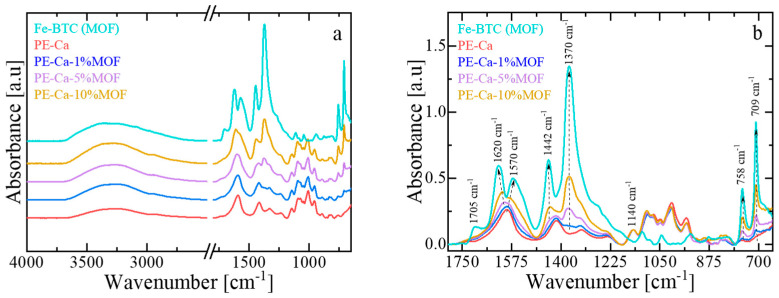
(**a**) FT–IR spectra of PE–Ca and PE–Ca–MOF composites containing water-dispersed Fe–BTC. (**b**) Detailed view of the PE and MOF carboxylate and MOF aromatic bands associated with the BTC linker. All spectra were normalized to the area of the glycosidic ring vibration (~1140 cm^−1^, –C–O–C) of the polygalacturonic acid backbone. Dashed arrows serve as visual guides to highlight the main spectral changes resulting from the incorporation of Fe–BTC into the pectin matrix.

Figure 3a,b shows the infrared spectra of Fe–BTC, PE–Ca, and the composites. Fe–BTC exhibits characteristic vibrational bands associated with the benzene–1,3,5–tricarboxylate (BTC) linkers (~1350–1650 cm^−1^) and Fe–oxo clusters (~680–780 cm^−1^) [16]. Strong asymmetric and symmetric O–C–O stretching vibrations, observed between ~1350–1650 cm^−1^, indicate deprotonated carboxylate groups coordinated to Fe^3+^ centers. Bands at around 1616 cm^−1^, 1450 cm^−1^ are attributed to carboxylate C=O and aromatic C=C stretching, respectively [17]. A weak band near 1700–1705 cm^−1^ is linked to uncoordinated –COOH groups, while a broad band at ~3300 cm^−1^ corresponds to O–H stretching from residual adsorbed water. PE–Ca shows a broad and intense band for –OH stretching vibrations (3600–3000 cm^−1^), and peaks around ~1725 cm^−1^ assigned to the C=O stretching of esterified or non-ionized carboxyl groups (–COOH, –COOCH_3_). Symmetric and asymmetric COO^−^ stretching vibrations appear near ~1400–1440 cm^−1^ and ~1585–1600 cm^−1^, respectively [24,25,26]. The FT–IR spectra of the pectin composites exhibit features from both PE–Ca and Fe–BTC, indicating that the MOF is physically immobilized within the pectin matrix without framework collapse. The characteristic framework bands (~1350–1650 cm^−1^ and ~680–780 cm^−1^) remain unchanged in the PE–Ca–MOF composites, showing that the phases are physically mixed with negligible interfacial interaction. The retention of these bands from each component suggests structural compatibility and confirms that no significant chemical reactions occurred during composite fabrication. The same conclusions were confirmed also from the XRD patterns of Fe–BTC, PE–Ca, and PE–Ca–5%MOF (see Section S4 in SM).

**Figure 4:**
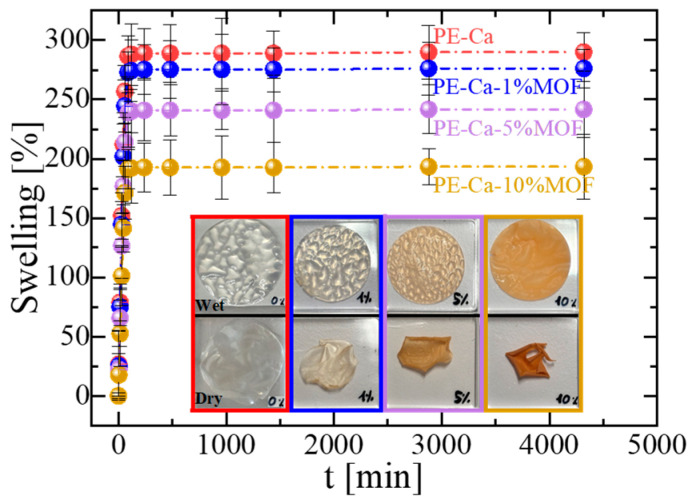
Swelling behavior of PE–Ca and PE–Ca–MOF composites containing water-dispersed Fe–BTC.

Figure 4 shows the effect of MOF amount on the swelling behavior of the composites; a higher swelling capacity facilitates the adsorbate diffusion into the matrix, increasing access to active sites and improving adsorption performance. While all composites reached swelling equilibrium within 90 min, increasing the amount of MOF led to reduced swelling, which also introduced brittleness. At 10 wt% of MOF, visible cracks appeared upon drying (see inset in Figure 4).

### 3.3. Influence of MOFs on the Adsorption of Heavy Metals

Pectin is widely recognized as an excellent adsorbent for heavy metals [50]. Our goal here was to determine if the composites containing MOFs retain this property by studying the adsorption of zinc ions (Zn^2+^). To isolate the contributions of each component, we first conducted adsorption experiments using pure MOFs powder, confirming its inactivity to adsorb Zn^2+^ ions. After that, we have investigated whether the presence of immobilized MOF influences the Ca^2+^/Zn^2+^ ion exchange within the PE matrix. Adsorption experiments with Zn^2+^ were conducted at an initial concentration of 50 mg/L, using a 1 g/L composite dose at pH 7 for 24 h for all adsorbents and compared with the reference PE–Ca (no MOF) (Table 2). As the MOF content increased, no significant differences in adsorption capacity were detected, confirming that the MOFs immobilization does not affect the adsorption properties of the PE–Ca.

**Figure 5:**
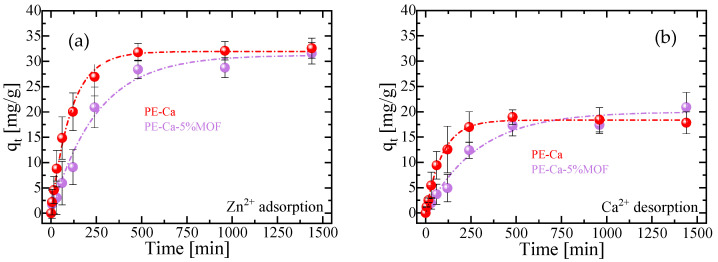
Kinetic profiles at pH = 7 of (**a**) adsorption of Zn^2+^ and (**b**) desorption of Ca^2+^ for PE–Ca and PE–Ca–5%MOF at 25 °C, using a composite dose of 1 g/L (C_0_ = 50 mg/L). The fits to the PFO model are represented by dashed lines.

This finding is critical, as it allows us to conclude that any heavy metal adsorption detected in the PE–Ca–MOF composites is solely attributable to the pectin matrix. Kinetic analysis of zinc adsorption on PE–Ca and PE–Ca5%MOF, performed with an initial zinc concentration of 50 mg/L and a composite dose of 1 g/L at pH 7, is shown in Figure 5a. The kinetic data are well described by the pseudo-first-order (PFO) model, indicating that Zn^2+^ binding is mainly governed by electrostatic interactions [31,43], with fitting parameters detailed in Table 3. The addition of the MOF slows the adsorption rate, as indicated by the lower k_1_ value in PE–Ca–5%MOF compared to PE–Ca. This decreased kinetic rate and longer time to reach equilibrium are likely a result of the reduced swelling of the composite (Figure 4). Furthermore, the q_eq_ value is consistent for both samples, indicating that the presence of Fe–BTC does not affect the adsorption of heavy metals on pectin.

To confirm the underlying adsorption mechanism, the kinetics of calcium desorption were simultaneously measured with the zinc uptake, as shown in Figure 5b. The Ca^2+^/Zn^2+^ exchange ratio was close to 1 in all cases (see Table 3). This demonstrates that the adsorption mechanism remains an ion exchange process; however, the presence of the MOF in the composite noticeably slows the kinetics of this process, which is attributable to its reduced swelling capacity relative to the reference PE–Ca (Figure 4). Since Zn^2+^ is also able to crosslink low-methoxyl pectin, the Ca^2+^-to-Zn^2+^ ion exchange during adsorption is expected to convert Ca–pectin junctions into Zn–pectin junctions rather than decrosslinking the network, consistent with the preserved macroscopic integrity of the films after Zn^2+^ uptake [31]. Finally, ICP measurements of the equilibrium solutions did not detect iron, confirming that the MOF particles are not released into the aqueous phase under these conditions.

In these composites, the degree of swelling decreases as the MOF content increases (Figure 4), consistent with the more rigid character of the network at higher Fe–BTC loadings, which increase T_g_ (Figure 2b). Adsorption then proceeds more slowly than swelling because, beyond simple water uptake, the contaminants must diffuse through the hydrated network, locate specific active sites, and interact with the corresponding functional groups, which requires at least partial disruption of their hydration shells. At short contact times, when swelling is still limited, the pores remain more constricted and partially hinder access of Zn^2+^ and other molecules to the internal adsorption sites, whereas once the material is fully swollen and the network is more open, the adsorption process is markedly enhanced. Under these conditions, adsorption equilibrium is reached after ap–proximately 6–8 h, reflecting the additional diffusive and interfacial steps involved compared with pure hydration

### 3.4. Effect of MOFs Content on the Adsorption of Pharmaceuticals and Pesticides

**Figure 6:**
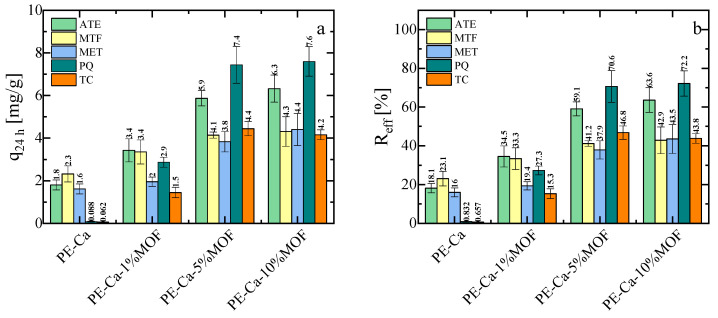
(**a**) Adsorption capacity and (**b**) removal efficiency of PE–Ca and PE–Ca composites with water-dispersed Fe–BTC after 24 h at an initial concentration of 10 mg/L, at a composite dose of 1 g/L and at pH 7.

To evaluate the potential of the composite material, we tested its adsorption capacity for a range of contaminants at pH 7. It is important to note that PE–Ca films (no MOF) can adsorb divalent and trivalent heavy metals as well as zwitterionic and mixed positively charged and neutral molecules, including atenolol (ATE), metformin (MTF), and metoprolol (MET) [51]. However, PE–Ca films are ineffective against tetracycline (TC) or paraquat (PQ), due to their poor compatibility with the pectin network. Consistent with previous reports [52], strongly positively charged molecules such as PQ can show negligible adsorption on PE–Ca, because the tightly bound hydration shell around the cationic adsorption sites makes dehydration and approach to the carboxylate sites energetically unfavorable; analogous behavior has been observed for other highly hydrated cationic species. In contrast, Fe–BTC MOF in powder form is highly effective in removing these contaminants (removal efficiency > 90%) (See Section S5 in SM) [18] because of its porous aromatic structure, which allows multiple non-covalent interactions, including electrostatic and π–π stacking attraction [13,18,19]. Figure 6a,b show the adsorption capacity and removal efficiency of ATE, MET, MFT, PQ, and TC at an initial concentration of 10 mg/L, using a 1 g/L composite dose at pH 7 for 24 h. As expected, the PE–Ca film showed no adsorption of PQ or TC after 24 h. However, the addition of Fe–BTC significantly improves the adsorption performance for all the contaminants (except for Zn, whose adsorption results are similar and independent of Fe–BTC content, as previously discussed in Section 3.2). While the best results are observed for the pectin filled with 10 wt% MOF, this sample has large aggregates and is very brittle, making it unsuitable for water remediation. Consequently, the sample with 5 wt% MOF content was selected for further studies on isotherms, kinetics, and reusability. In the adsorption experiments with organic contaminants, the supernatants were additionally monitored by UV–Vis spectroscopy, and no Fe–related bands or spectral shifts attributable to Fe–contaminant complexes were observed, further confirming that the Fe–BTC phase remained effectively immobilized within the pectin matrix during operation (see Section S6 in SM). The same conclusion was reached for the reusability tests after mild acidic regeneration over multiple cycles (Section 3.7).

### 3.5. Kinetics and Isotherms of Adsorption of Paraquat and Tetracycline on PE–Ca–5%MOF

**Figure 7:**
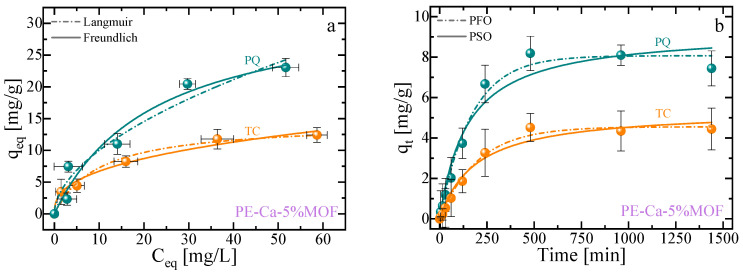
Adsorption isotherms (**a**) and adsorption kinetics (**b**) for paraquat (PQ) and tetracycline (TC) for PE–Ca–5%MOF at a composite dose of 1 g/L and pH 7. Isotherms performed at an initial concentration ranging from 5 to 75 mg/L and fitted using the Langmuir (dashed lines) and Freundlich (solid lines) models. Kinetics performed at an initial concentration of 10 mg/L and fitted using the PFO (dashed lines) and PSO (solid lines) models.

Figure 7a,b show the adsorption isotherms and kinetics profiles for both contaminants. Although a total composite dose of 1 g/L was used, the calculated adsorption capacities were normalized by the mass of the active sites in the PE–Ca–5%MOF composite. This was possible because the PE–Ca matrix is not able to adsorb the PQ and TC (Figure 6). The UV–Vis spectra of PQ and TC stock solutions, respectively, used for adsorption isotherms analysis (initial concentration of 5, 10, 25, 50 and 75 mg/L) are reported in the Section S5 in SM. The isotherms data (Figure 7a) were best described by the Langmuir model [44,53], which assumes that adsorption at identical sites with a monolayer coverage (only one molecule can be adsorbed at each active site); the fitting results for both models are reported in Table 4.

Figure 7b shows the adsorption kinetics of PQ and TC as a function of time (initial concentration of 10 mg/L, pH 7). Both contaminants are better fitted with a pseudo-first-order (PFO) kinetics model, indicating that Van der Waals interactions play a significant role in the adsorption mechanism. Notably, PQ was adsorbed more rapidly than TC, likely due to its structure allowing for more efficient π−π stacking interaction. The statistical fitting results for both the isotherms and kinetics studies are summarized in Table 4 and Table 5, respectively.

### 3.6. Interaction Mechanism

**Figure 8:**
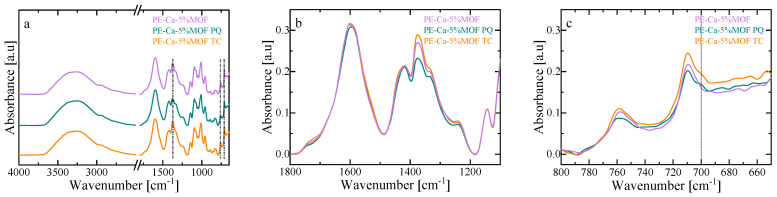
FT–IR spectra of Fe–BTC before and after adsorption of paraquat (PQ) and tetracycline (TC): (**a**) full spectral range, (**b**) carboxylate, and (**c**) aromatic regions (~750–1650 cm^−1^). The black dashed line indicates the wavenumber 700 cm^−1^.

The interaction between the adsorbent and PQ/TC was investigated using Fourier transform infrared (FT-IR) spectroscopy. As shown in Figure 8a, Fe–BTC keeps its structural integrity after adsorption, as its characteristic vibrational bands of the BTC linker remain visible (carboxylate stretches (~1350–1650 cm^−1^) and Fe–oxo bridge (~680–780 cm^−1^). The presence of N–H and C–H shoulders in the 2800–3000 cm^−1^ region, together with a broadening of the O–H band near 3300 cm^−1^, is consistent with the formation of hydrogen bonds between the contaminants and the material, and is more pronounced for TC due to its additional hydroxyl and amide groups [20]. Adsorption of both TC and PQ also leads to changes in the free C=O stretching band near ~1700 cm^−1^ (Figure 8b) and to slight shifts in the carboxylate region (1350–1650 cm^−1^), suggesting partial deprotonation and coordination with unsaturated Fe^3+^ sites, forming Fe–OOC–TC/PQ linkages [17,54]. Subtle modifications in the ~680–780 cm^−1^ region are compatible with additional electrostatic and polar interactions between PQ or TC and Fe–oxo bridges (Figure 8c). Moreover, PQ and TC may interact through π–π stacking between their aromatic rings and the BTC linker [54], accompanied by band shifts and the appearance of a shoulder around 700 cm^−1^ that is compatible with the formation of π donor–acceptor complexes [17]. Overall, the observed shifts and broadenings of the Fe–BTC bands (Figure 8c) are consistent with effective adsorption of both contaminants through non-covalent interactions. Within this picture, TC appears to interact predominantly via coordination to Fe^3+^, hydrogen bonding and π–π stacking [55], whereas PQ seems to interact mainly through π–π donor–acceptor interactions with the BTC linker together with weaker electrostatic contributions [17].

### 3.7. Reusability of PE–Ca–5%MOF

**Figure 9:**
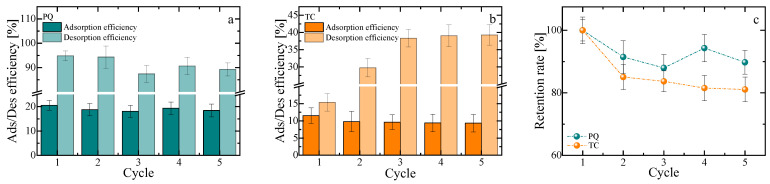
Adsorption–desorption efficiency of (**a**) paraquat (PQ) and (**b**) tetracycline (TC) over five consecutive cycles for PE–Ca–5%MOF. Reusability tests were performed with a fixed contact time of 1 h for both adsorption and desorption steps, using an initial adsorbate concentration of 10 mg/L, a composite dose of 1 g/L, and pH 7 for all cycles. (**c**) Retention rate of PQ and TC over the five reusability cycles.

Figure 9a,b show the results over five adsorption–desorption cycles for PE–Ca5%MOF with both pollutants, PQ and TC. The results indicate that the adsorbent can be reused after desorption with no significant decrease in performance, demonstrating its suitability for industrial uses. An acidic solvent was chosen as a desorption medium because this method is inexpensive, quick, and environmentally friendly.

The results showed that the composite performed significantly better for PQ compared to TC after the first adsorption–desorption cycle, indicating a stronger interaction between TC and the active sites of the composites. Conversely, PQ adsorption–desorption showed no significant changes during the cycles, confirming a weakly bound interaction that facilitates its release under acidic conditions. However, TC, likely due to its heavier and more complex structure and additional functional group interactions, is less desorbed under the same conditions.

As shown in Figure 9c, PQ retains (87.9 ± 4.3) % of its initial capacity, whereas TC declines to (81.1 ± 3.5) % by the fifth cycle, suggesting partial occupation of irreversible sites after desorption. Overall, PE–Ca–5%MOF demonstrates better reusability for PQ than for TC under the tested conditions. The retention rate was defined as the ratio between the adsorption capacity in cycle n and that of the first cycle for the PE–Ca–5%MOF composite, with PE–Ca acting only as a structural blank (it shows negligible uptake and is not an active adsorbent for the studied contaminants), so that the reported values should be interpreted as apparent capacity retention under the specific adsorption–desorption protocol rather than as full proof of long-term regenerability. In addition, Fe-leaching data obtained by ICP and UV–Vis show no detectable iron release during adsorption–desorption, supporting the effective immobilization of the Fe–BTC phase in the pectin matrix under the conditions used.

### 3.8. Structural Characterization of PE–Ca–MOF Film and Foam Using the PVP–Dispersed Method

While incorporating MOFs into a pectin matrix significantly enhances the capacity of the adsorbent, the current approach results in poor dispersion of the MOF, as illustrated in Figure 1. This inadequate dispersion is problematic because it prevents the composite from realizing its full potential. When MOF particles agglomerate, their surface area for interaction decreases, thereby reducing the number of active sites necessary for adsorption. Consequently, enhancing dispersion is essential to boost adsorption performance. Several strategies can be employed to improve dispersion, such as surface modification of the MOF particles, using high-shear mixing during the composite fabrication, or enhancing MOF dispersion with surfactants that adsorb onto the surface of the particles and create a steric hindrance that prevents agglomeration [46]. Additionally, these approaches can help lower the MOF concentration, reducing the final cost of the adsorbent.

We explored two different strategies to improve the dispersion of MOF. In the first approach, we have used polyvinylpyrrolidone (PVP) as a surfactant, which prevents MOF from agglomerating (PE–Ca–1%MOF–PVP film) [46]. For the second strategy, apart from adding the PVP, the shape of the pectin matrix was modified by forming a highly porous structure (foam) through freeze-drying, which increased the surface area of the material, allowing for better exposure to contaminated water (PE–Ca–1%MOF–PVP foam). ICP–AES results indicate that neither the dispersion method nor the composite morphology significantly affects calcium adsorption during crosslinking or zinc adsorption during removal.

**Figure 10:**
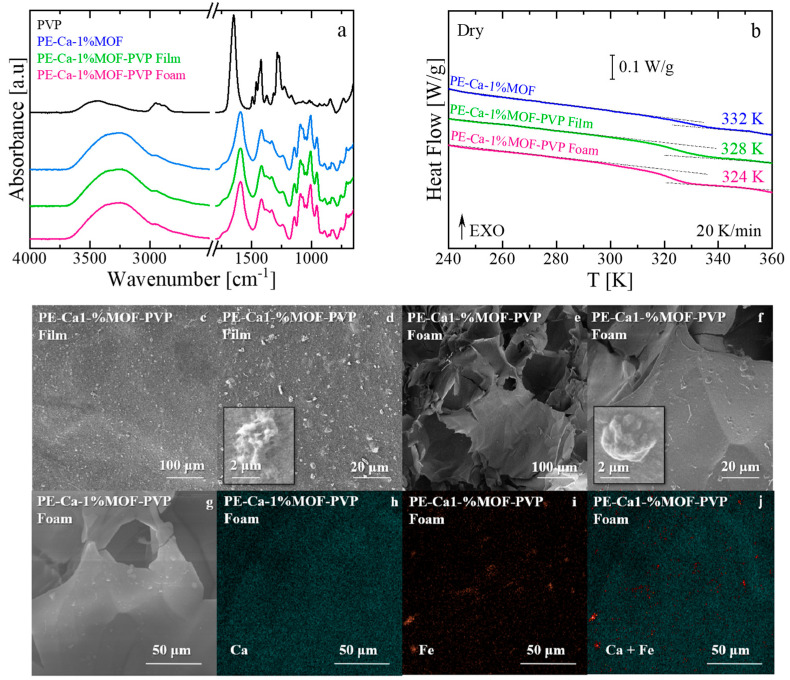
(**a**) Infrared spectra and (**b**) Heat flow versus temperature after thermal aging of PE–Ca–1%MOF (water-dispersed), PE–Ca–1%MOF–PVP film, and PE–Ca–1%MOF–PVP foam. The *T_g_* is marked in the figure. The arrow EXO means that any exothermic event (releasing heat) is shown as an upward peak or curve. (**c**–**f**) SEM images of PVP–assisted 1 wt% MOF dispersion in film (**c**,**d**) and foam (**e**,**f**). (**g**–**j**) EDAX elemental mapping of calcium and iron for the corresponding foam samples.

As shown in Figure 10, the structural characteristics of the samples containing a surfactant (PE–Ca–1%MOF–PVP film) and after freeze-drying (PE–Ca–1%MOF–PVP foam) were compared to the water-dispersed (PE–Ca–1%MOF) and pure dry PVP control samples. Infrared spectroscopy (Figure 10a) revealed no changes in the characteristic bands of either the MOF or the pectin after adding PVP or forming the foam, indicating that the chemical structures of all components remain unchanged after improving the dispersion of Fe–BTC. The FT–IR spectra of the rinsed composites do not show the characteristic bands of PVP, indicating that PVP is largely removed during the washing steps, and that any direct contribution of PVP to the adsorption performance of the final composites is negligible.

DSC analysis nevertheless reveals a marked decrease in T_g_ for PE–Ca–1%MOF–PVP relative to the water dispersed PE–Ca–1%MOF control (Figure 10b), which we attribute to the transient porogen-like action of PVP during processing; when the PVP–MOF solution is mixed with the pectin prior to crosslinking and rinsing, (Section 2.2), PVP temporarily plasticizes the pectin network, improves MOF dispersion, and promotes the formation of pores and voids, thereby increasing the free volume. This effect is further amplified in the foam sample, which exhibits a larger T_g_ decrease due to the formation of pores and voids within its structure, thereby increasing the free volume between polymer chains [56,57]. After crosslinking and washing, the reduced T_g_ is thus attributed to this porogen-mediated increase in free volume rather than to the presence of PVP as a retained plasticizer in the final composite.

The structural difference between the sample made with the surfactant and those made in a foam shape is evident in the SEM images shown in Figure 10c–f. Compared to the film (Figure 10a,b), the foam architecture (Figure 10c,d) exhibits a significantly larger active area due to its high porosity. Furthermore, adding the surfactant creates a more homogeneous dispersion of the MOF solution (also confirmed by DLS measurements, see Section 3.1), in line with better particle distribution in both architectures (film and foam) compared to the water-dispersed method used to make the film shown in Figure 1. The corresponding EDX maps (Figure 10g–j) further suggest this improvement by showing a more even distribution of calcium and iron throughout the pectin matrix, with smaller and thinner aggregates compared to the larger clusters seen in water-dispersed composites (Figure 1). The SEM and EDX images should be regarded as representative examples that point to an improvement in Fe–BTC dispersion in the PVP assisted samples, rather than as proof of a perfectly uniform distribution. These techniques provide local, mainly qualitative information and do not yield a full statistical mapping of particle positions. When considered together with the DLS data, however, they support an overall improvement in MOF dispersion in the presence of PVP.

**Figure 11:**
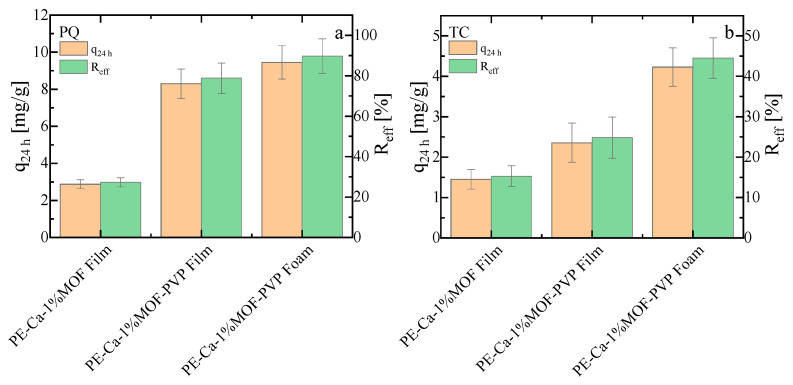
Adsorption capacity and removal efficiency of (**a**) paraquat (PQ) and (**b**) tetracycline (TC) for PE–Ca composites prepared with 1 wt% of MOF loading and dispersion methods (water or PVP-assisted, in film and foam geometries). Tests were conducted at an initial concentration of 10 mg/L, composite dose of 1 g/L, and contact time of 24 h at pH 7.

The adsorption capacity and removal efficiency of the PVP-assisted film and foam containing 1 wt% of MOF were compared with those of the water-dispersed control sample (PE–Ca–1%MOF) for PQ (Figure 11a) and TC (Figure 11b), respectively. These experiments were conducted at an initial concentration of 10 mg/L, with a composite dose of 1 g/L, over 24 h at pH 7. The PVP-assisted film and foam, due to its better distribution of smaller MOF particles and porous structure with larger surface area, performed better than the water-dispersed samples (PE–Ca–1%MOF) (see Section S6 in SM for UV–Vis spectra for comparison of composites with 1 wt% and 5 wt% water dispersed MOF and PVP-dispersed films and foams with 1 wt% of MOF). Since TC is a larger and heavier molecule, the foam structure allows easier access to the MOF particles, resulting in higher adsorption performance. These findings demonstrate that using PVP as a dispersant agent enables the use of less MOF quantity in the composite improving the adsorption performance. The PVP-assisted foam shows the highest adsorption performance in comparative batch tests (Figure 11); however, detailed isotherm and kinetic analyses are still needed to fully validate the mechanistic interpretation established for the water-dispersed composites.

At realistic pollutant concentrations (1 mg/L of Zn^2+^, PQ, and TC each), simultaneous treatment leads to essentially quantitative removal, reaching efficiencies of 100 ± 1% within a few minutes.

Because the present adsorbent is a pectin-supported MOF composite, while most of the literature capacities are reported for neat powders under different loadings, direct quantitative comparison of q per gram of material is not straightforward; therefore, we limit our analysis to the raw experimental capacities per gram of composite and to a qualitative discussion of how these values relate to typical MOF powders.

## 4. Conclusions

This study successfully developed and characterized sustainable, bio-based adsorbents by integrating the metal-organic framework Fe–BTC MOF within a calcium-crosslinked pectin matrix, systematically demonstrating the critical roles of fabrication parameters in enhancing water purification performance. Optimal dispersion, achieved through a poly(vinylpyrrolidone) (PVP-assisted method), significantly reduced MOF particle aggregation, leading to a better distribution within the polymer and a notable improvement in overall adsorption capacity. Furthermore, the geometry of the materials was shown to be a key determinant of performance, with freeze-dried foams exhibiting superior porosity and faster adsorption kinetics compared to solvent-cast films. Freeze-dried foams exhibited higher porosity and higher adsorption capacity compared to solvent-cast films due to enhanced mass transfer through interconnected porous networks and improved accessibility of active sites.

The water-dispersed PE–Ca–MOF films exhibit higher equilibrium capacities than PE–Ca but a lower pseudo-first-order rate constant, in line with their reduced swelling and increased rigidity, which slows contaminant transport to the active sites. The PVP-assisted foam shows the highest uptake in comparative batch experiments, likely due to its open-cell structure and shorter diffusion paths; however, a complete kinetic analysis analogous to that performed for the water-dispersed films remains to be carried out to characterize its adsorption rates quantitatively.

This finding provides design principles for optimizing adsorbent geometry based on application requirements (e.g., batch vs. flow-through systems). The resultant pectin-MOF composite demonstrated versatile and effective removal of a range of water contaminants, including a heavy metal (Zn^2+^), a pesticide (paraquat), and an antibiotic (tetracycline). This broad-spectrum efficiency is attributed to the complementary binding mechanisms resulting from the synergy between the pectin carboxyl groups and the coordination sites of the MOF. Analyses revealed that the adsorption process is best described by diffusion-controlled pseudo-first-order kinetics and fits the Langmuir isotherm model, confirming indicating favorable and largely homogeneous adsorption. Importantly, the composites exhibited good practical reusability, maintaining high capacity over multiple cycles when regenerated using a bio-based 2% *v*/*v* acetic acid solution, thus confirming their structural stability and viability for repeated environmental applications.

Finally, although the exact MOF loading in the final composites was not independently quantified by acid digestion, multiple independent observations support effective retention of the Fe–BTC phase within the pectin matrix: the absence of detectable Fe^3+^ in all aqueous phases (ICP), the co–localization of iron with MOF-containing regions in EDX mapping, and the systematic increase in adsorption capacity with nominal MOF loading. Consequently, all reported adsorption capacities are expressed per gram of composite material and do not depend on assumptions about the exact MOF content. All experiments were conducted at neutral pH (pH 7) using ultrapure water and single contaminants; the pH-dependent behavior of these contaminants and the effects of competing ions or natural organic matter typical of real-world water matrices remain to be investigated in future work.

## Figures and Tables

**Table 1:** Calcium content (in mg/L and mmol) measured by ICP–AES and calculated stoichiometric ratio (R) for PE–Ca samples with various MOF contents after five washing cycles.

Material	Ca^2+^_ads_ [mg/L]	Ca^2+^_ads_ [mmol]	R
PE–Ca	67.5 ± 2.9 ^a^	1.69 ± 0.07 ^a^	0.29
PE–Ca–1%MOF	67.5 ± 1.6 ^a^	1.68 ± 0.04 ^a^	0.29
PE–Ca–5%MOF	68.6 ± 2.7 ^a^	1.71 ± 0.07 ^a^	0.29
PE–Ca–10%MOF	66.2 ± 3.1 ^a^	1.65 ± 0.08 ^a^	0.28

^a^ Values with the same letters in the columns are not significantly different (errors arise from the experimental standard deviation of triplicate measurements).

**Table 2:** Zn^2+^ adsorption determined by ICP–AES using a composite dose of 1 g/L and a 50 mg/L of initial Zn^2+^ concentration for 24 h. The table reports the Zn^2+^ adsorption capacity (q_24 h_) and removal efficiency (R_eff_) of PE–Ca–MOF composites.

Zn^2+^ Adsorption		
Material	q_24h_ [mg/g]	R_Eff_ [%]
PE–Ca	31.9 ± 1.9 ^a^	63.9 ± 3.9 ^a^
PE–Ca–1%MOF	31.8 ± 2.4 ^a^	62.4 ± 4.7 ^a^
PE–Ca–5%MOF	31.6 ± 2.1 ^a^	62.1 ± 4.2 ^a^
PE–Ca–10%MOF	32.5 ± 2.9 ^a^	63.9 ± 5.6 ^a^

^a^ Values with the same letters in the columns are not significantly different (errors arise from the experimental standard deviation of triplicate measurements).

**Table 3:** Ca^2+^ desorption and Zn^2+^ adsorption kinetics of PE–Ca and PE–Ca–5%MOF films determined by ICP–AES, when experiments are performed at pH = 7, dose of 1 g/L, and an initial Zn^2+^ concentration (C_0_) of 50 mg/L. Kinetic data were fitted using the PFO model. The table reports the correlation coefficient (R^2^), the kinetic constant (k_1_ [min^−1^]), the equilibrium adsorption capacity (q_e_), the removal efficiency (R_E_), and the Ca^2+^/Zn^2+^ exchange ratio.

		Zn^2+^ Adsorption				Ca^2+^ Desorption			
Material	R^2^	k_1_ Zn^2+^ [min^–1^]	q_e_ Zn^2+^ [mg/g]	Zn^2+^ [mmol]	R_Eff_ [%]	k_1_ Ca^2+^ [min^–1^]	q_e_ Ca^2+^ [mg/g]	Ca^2+^ [mmol]	Exchange Ratio
PE–Ca	0.99	0.0091 ± 0.0005 ^a^	31.9 ± 0.6 ^a^	0.48 ± 0.01 ^a^	63.8 ± 1.2 ^a^	0.0109 ± 0.0006 ^a^	18.4 ± 0.3 ^a^	0.46 ± 0.01 ^a^	1.04 ± 0.03 ^a^
PE–Ca–5%MOF	0.99	0.0040 ± 0.0004 ^b^	31.2 ± 1.2 ^a^	0.48 ± 0.02 ^a^	62.4 ± 2.4 ^a^	0.0035 ± 0.0005 ^b^	19.9 ± 0.9 ^b^	0.49 ± 0.02 ^a^	0.98 ± 0.06 ^a^

^a,b^ Values with the same letters in the columns are not significantly different (errors correspond to the uncertainties obtained from the model fits; *p* > 0.01).

**Table 4:** Statistical adsorption isotherms fitting results for paraquat (PQ) and tetracycline (TC).

Adsorption Isotherms								
	Langmuir				Freundlich			
Pollutant	R^2^	χ^2^	q_m_ [mg/g]	K_L_ [L/mg]	R^2^	χ^2^	K_F_ [L^1/n^mg^1−1/n^g^−1^]	1/*n*
PQ	0.95	4.8	33.5 ± 6.9 ^a^	0.05 ± 0.02 ^a^	0.91	6.8	2.9 ± 1.2 ^a^	0.5 ± 0.1 ^a^
TC	0.97	0.6	14.5 ± 1.3 ^b^	0.10 ± 0.03 ^a^	0.96	0.8	2.8 ± 0.5 ^a^	0.37 ± 0.05 ^a^

^a,b^ Values with the same letters in the columns are not significantly different (errors correspond to the uncertainties obtained from the model fits; *p* > 0.01).

**Table 5:** Statistical adsorption kinetics fitting results for paraquat (PQ) and tetracycline (TC).

Adsorption Kinetics								
	PFO				PSO			
Pollutant	R^2^	χ^2^	q_m_ [mg/g]	k_1_ [min^−1^]	R^2^	χ^2^	q_m_ [mg/g]	K_2_ [min^−1^]
PQ	0.99	0.2	8.1 ± 0.3 ^a^	0.0059 ± 0.0006 ^a^	0.96	0.5	9.3 ± 0.7 ^a^	0.00073 ± 0.00023 ^a^
TC	0.99	0.03	4.6 ± 0.1 ^b^	0.0049 ± 0.0004 ^a^	0.97	0.1	5.4 ± 0.4 ^b^	0.00098 ± 0.00027 ^a^

^a,b^ Values with the same letters in the columns are not significantly different (errors correspond to the uncertainties obtained from the model fits; *p* > 0.01).

## Data Availability

The original contributions presented in this study are included in the article/Supplementary Material. Further inquiries can be directed to the corresponding authors.

## Supplementary Materials

The following supporting information can be downloaded at: https://www.mdpi.com/article/10.3390/polym18020171/s1, Table S1: Statistical parameters for the PFO model fits in Figure 5 for Zn2+ adsorption and Ca2+ desorption; Table S2: Statistical parameters for the Langmuir and Freundlich isotherms fit in Figure 7 for PQ and TC adsorption; Table S3: Statistical parameters for the PFO and PSO kinetics fit in Figure 7 for PQ and TC adsorption; Table S4: Akaike Information Criterion (AIC) and Bayesian Information Criterion (BIC) values for the isotherm and kinetics fits shown in Figure 7 for both PQ and TC contaminants. Lower AIC/BIC values indicate the preferred model; Table S5: Statistical parameters of the calibration curves used for quantification of paraquat (PQ) and tetracycline (TC); Figure S1: FT–IR baseline correction and normalization; Figure S2: N2 adsorption isotherms at −196 °C on commercial MOF Basolite F300® (Fe–BTC); Figure S3: XRD patterns of pure Fe–BTC, PE–Ca, and PE–Ca–5%MOF composite. The inset highlights the diffraction region between 5 ° and 20 °, showing the combined structural features of the MOF and the polymer matrix; Figure S4: Removal efficiency pure Fe–BTC (MOF) powder after 24 h at an initial concentration of 10 mg/L, at a composite dose of 1 g/L and at pH 7; Figure S5: UV–Vis spectra of (a) paraquat (PQ) and (b) tetracycline (TC) at initial concentrations ranging from 5 to 75 mg/L before (solid lines) and after 24 h of adsorption by PE–Ca–5%MOF (dashed lines) at a composite dose of 1 g/L and pH 7. Insets show the calibration curves constructed from the maximum absorbance values at 260 nm for PQ and 356 nm for TC; Figure S6: UV–Vis spectra of (a) paraquat (PQ) and (b) tetracycline (TC) at an initial concentration of 10 mg/L after 24 h of adsorption (composite dose: 1 g/L, pH 7) by PE–Ca composites containing 1 wt% and 5 wt% Fe–BTC (water-dispersed) and 1 wt% Fe–BTC (PVP-assisted) in film and foam geometries. Reference [58] is cited in Supplementary Materials.
