# Supplementary material for: Bio-Based Pectin-Calcium Film and Foam Adsorbents with Immobilized Fe–BTC MOF for Water Contaminant Removal
Source: Polymers (Basel). 2026 Jan 8;18(2):171. doi: 10.3390/polym18020171 (PMC12845238; doi:10.3390/polym18020171)
Supplement: Supplementary file 1 [file polymers-18-00171-s001.zip › polymers-4041095-supplementary.docx]

Supplementary Materials

Bio–Based Pectin–Calcium Film and Foam Adsorbents with Immobilized Fe–BTC MOF for Water Contaminant Removal

Francesco Coin ^1,^*, Carolina Iacovone ^1,2^ and Silvina Cerveny ^1,^*

^1^ *Centro de Física de Materiales (CSIC, UPV/EHU)–Materials Physics Center (MPC), Paseo Manuel de Lardizabal 5, San Sebastián 20018, Spain*

^2^ *Donostia International Physics Center (DIPC), Paseo Manuel de Lardizabal 4, San Sebastián 20018, Spain*

***** Correspondence: fcoin001@ikasle.ehu.eus (F.C.); silvina.cerveny@ehu.es (S.C.)

**Keywords:** bio–based adsorbents; metal–organic framework (MOF); water purification; pectin–calcium films and foams; iron–based–MOF; adsorption

S1. Statistical Parameters and Model–Selection Criteria for Adsorption Isotherm and Kinetic Models

The following tables (Tables S1–S3) summarize the statistical descriptors used to evaluate the isotherm and kinetic adsorption data of paraquat (PQ) and tetracycline (TC). “Points number” indicates how many experimental measurements were included in the regression, while the “Degree of freedom” reflects the amount of independent information remaining after estimating the model parameters. The “Residual sum of squares (RSS)” quantifies the overall discrepancy between the experimental data and the model predictions, and the χ^2^ value provides an additional goodness–of–fit metric. In both cases, lower values indicate a closer agreement with the experimental profile. The “t–Value” columns for q_e_​ and k_1__report the statistical strength of each fitted parameter relative to its uncertainty, whereas “Prob>|t|” gives the associated two–sided p–values; small p–values indicate that q_e_​ and k_1_​ are statistically significant and meaningfully contribute to describing the adsorption kinetics.

**Table S1.** Statistical parameters for the PFO model fits in Figure 5 for Zn^2+^ adsorption and Ca^2+^ desorption.

| **Zn^2+^ Adsorption** | | | | | | | | |  |
| --- | --- | --- | --- | --- | --- | --- | --- | --- | --- |
| **Material** | **χ^2^** | **Points number** | **Degree of freedom** | **Residual sum of squares (RSS)** | **t–Value q_e_** | **t–Value k_1_** | **Prob>\|t\| q_e_** | **Prob>\|t\| k_1_** | |
| PE–Ca | 0.9 | 10 | 8 | 7.8 | 56 | 15.7 | 1.1×10^–11^ | 2.6×10^–7^ | |
| PE–Ca–5%MOF | 2.4 | 10 | 8 | 19.4 | 26.6 | 8.8 | 4.2×10^–9^ | 2.1×10^–5^ | |
| **Ca^2+^ Desorption** | | | | | | | | | |
| **Material** | **χ^2^** | **Points number** | **Degree of freedom** | **Residual sum of squares (RSS)** | **t–Value q_e_** | **t–Value k_1_** | **Prob>\|t\| q_e_** | **Prob>\|t\| k_1_** | |
| PE–Ca | 0.3 | 10 | 8 | 2.1 | 65.3 | 17.9 | 3.4×10^–12^ | 9.8×10^–8^ | |
| PE–Ca–5%MOF | 1.4 | 10 | 8 | 11.1 | 20.9 | 7.2 | 2.8×10^–8^ | 9.3×10^–5^ | |

**Table S2.** Statistical parameters for the Langmuir and Freundlich isotherms fit in Figure 7 for PQ and TC adsorption.

| **Langmuir Isotherm** | | | | | | | | | | |  |
| --- | --- | --- | --- | --- | --- | --- | --- | --- | --- | --- | --- |
| **Material** | **χ^2^** | **Points number** | | **Degree of freedom** | **Residual sum of squares (RSS)** | **t–Value q_M_** | **t–Value K_L_** | | **Prob>\|t\| q_M_** | **Prob>\|t\| K_L_** | |
| PQ | 4.8 | | 6 | 4 | 19.1 | 4.8 | 5.1 | | 9×10^–3^ | 9×10^–3^ | |
| TC | 0.6 | | 6 | 4 | 3.4 | 10.9 | 3.2 | | 4×10^–4^ | 9×10^–3^ | |
| **Freundlich Isotherm** | | | | | | | | | | | |
| **Material** | **χ^2^** | **Points number** | | **Degree of freedom** | **Residual sum of squares (RSS)** | **t–Value K_F_** | | **t–Value 1/n** | **Prob>\|t\| K_F_** | **Prob>\|t\| 1/n** | |
| PQ | 6.9 | | 6 | 4 | 20.6 | 2.5 | 4.8 | | 8×10^–3^ | 9×10^–3^ | |
| TC | 0.8 | | 6 | 4 | 1.8 | 6.2 | 8.2 | | 9×10^–3^ | 4×10^–3^ | |

**Table S3.** Statistical parameters for the PFO and PSO kinetics fit in Figure 7 for PQ and TC adsorption.

| **PFO Kinetics** | | | | | | | | | |  |
| --- | --- | --- | --- | --- | --- | --- | --- | --- | --- | --- |
| **Pollutant** | **χ^2^** | **Points number** | **Degree of freedom** | **Residual sum of squares (RSS)** | **t–Value q_e_** | **t–Value k_1_** | | **Prob>\|t\| q_e_** | **Prob>\|t\| k_1_** | |
| PQ | 0.2 | 10 | 8 | 1.4 | 30.3 | 9.2 | | 1.5×10^–9^ | 1.5×10^–5^ | |
| TC | 0.03 | 10 | 8 | 0.3 | 35.7 | 11.3 | | 4.1×10^–10^ | 3.4×10^–6^ | |
| **PSO Kinetics** | | | | | | | | | | |
| **Pollutant** | **χ^2^** | **Points number** | **Degree of freedom** | **Residual sum of squares (RSS)** | **t–Value q_e_** | | **t–Value k_2_** | **Prob>\|t\| q_e_** | **Prob>\|t\| k_2_** | |
| PQ | 0.5 | 10 | 8 | 3.8 | 13.7 | 3.2 | | 7.8×10^–7^ | 9×10^–3^ | |
| TC | 0.1 | 10 | 8 | 0.8 | 15 | 3.6 | | 3.7×10^–7^ | 6×10^–3^ | |

The models used to fit the adsorption isotherms and kinetics were compared using R^2^ and reduced χ^2^ criteria. In addition, the models were evaluated based on the following criteria [1]:

– F–test;

– The Akaike Information criteria (AIC) [2];

– The Bayesian information criteria (BIC) [3].

Because the two candidate models for both the isotherm and kinetic datasets were fitted to the same data and had the same number of adjustable parameters, they had identical degrees of freedom; therefore, an F–test did not provide a meaningful basis for model discrimination and was inconclusive. Likewise, since the Langmuir and Freundlich isotherms and the PFO and PSO kinetic models were fitted using the same number of data points and parameters for both contaminants, the F–test remains inconclusive for these comparisons.

AIC and BIC are based on the likelihood function (Eq. 1 and Eq. 2 for AIC and BIC, respectively), and they are recognized as being able to measure the validity of non–linear models [1–3]. The lowest AIC or BIC value between the two compared models indicates the model that provides the best fit to the data..

$AIC=2p-2ln(L)$ (3)

$BIC=p\ln(n)-2\ln(L)$ (4)

where, *n* is the number of points in your data sample, p is the number of parameters estimated by the model, and *L* is the maximized value of the likelihood function of the model.

Table S4 reports the AIC and BIC values used to select the isotherm and kinetics model fits shown in Figure 7. In our case, the R^2^, reduced χ^2^, AIC, and BIC criteria all agree on the model that best fits the data. Among the isotherm models evaluated, the Langmuir equation provided the best fit for the adsorption of paraquat (PQ) and tetracycline (TC). The pseudo-first-order (PFO) model best described the kinetic data for both pollutants.

**Table S4.** Akaike Information Criterion (AIC) and Bayesian Information Criterion (BIC) values for the isotherm and kinetics fits shown in Figure 7 for both PQ and TC contaminants. Lower AIC/BIC values indicate the preferred model.

| **Isotherms** | | | | | | | |
| --- | --- | --- | --- | --- | --- | --- | --- |
| **Pollutant** | **Model** | **Points**  **number** | **Parameter number** | **F–test** | **AIC** | **BIC** | **Preferred model** |
| PQ | Langmuir | 6 | 2 | / | 24.9 | 11.9 | Langmuir |
|  | Freundlich | 6 | 2 | / | 37.1 | 12.3 |  |
| TC | Langmuir | 6 | 2 | / | 14.5 | –0.3 | Langmuir |
|  | Freundlich | 6 | 2 | / | 24.9 | 1.9 |  |
| **Kinetics** | | | | | | | |
| **Pollutant** | **Model** | **Points**  **number** | **Parameter number** | **F–test** | **AIC** | **BIC** | **Preferred model** |
| PQ | PFO | 10 | 2 | / | –7.1 | –10.2 | PFO |
|  | PSO | 10 | 2 | / | –1.9 | –4.9 |  |
| TC | PFO | 10 | 2 | / | –25.9 | –28.9 | PFO |
|  | PSO | 10 | 2 | / | –14.9 | –18.1 |  |

S2. FT–IR normalization

Figure S1 shows the FT–IR baseline correction and normalization considering the peak area of the glycosidic ring vibration of the poly–galacturonic acid chain (–C–O–C) at ~1140 cm^–1^ [4].

**Figure S1.** FT–IR baseline correction and normalization.

S3. Adsorption Isotherms of Nitrogen

Adsorption–desorption isotherms of nitrogen at −196 °C were recorded using the Micromeritics ASAP 2420 instrument. Before sorption measurement, the sample was degassed at a pressure below 10–4 mbar and 200 °C for 1 hour using nitrogen gas. The BET area was determined using the Brunauer–Emmett–Teller (BET) method, based on adsorption data in the p/p_0_ range of 0.05–0.20 (Figure S2). The volume of micropores (VMI) was evaluated using the DFT method (N_2_ adsorption considering cylindrical pores). Adsorption isotherms of nitrogen at 77 K reveal a pore diameter of 2.0 nm (nm) and a BET area of 1366 m^2^/g for Fe–BTC.

**Figure S2.** **(a)** N_2_ adsorption isotherms at −196 °C on commercial MOF Basolite F300^®^ (Fe–BTC). (b) BJH pore size distribution on commercial MOF Basolite F300^®^ (Fe–BTC).

S4. X–Ray Diffraction

The PE–Ca film shows a broad amorphous peak at 14.02 °, which emerges after Ca^2+^ crosslinking [5],[6]. Fe–BTC exhibits sharper diffraction peaks, with an amorphous background, typical of Fe–BTC structures [7]. The PE–Ca–5%MOF pattern displays combined features of both components (Figure S3). Notably, a shoulder appears at 14.02 °, near the main Fe–BTC peak at 11.11 ° (inset on Figure S3), indicating successful incorporation of the MOF into the pectin matrix without structural disruption or chemical incompatibility

**Figure S3.** XRD patterns of pure Fe–BTC, PE–Ca, and PE–Ca–5%MOF composite. The inset highlights the diffraction region between 5 ° and 20 °, revealing the combined structural features of the MOF and the polymer matrix.

S5. Fe–BTC contaminants adsorption

Figure S4 show the removal efficiency of ATE, MET, MFT, PQ and TC at an initial concentration of 10 mg/L, using a 1 g/L dose at pH 7 for 24 hours for Fe–BTC.

**Figure S4.** Removal efficiency pure Fe–BTC (MOF) powder after 24 h at an initial concentration of 10 mg/L, at a composite dose of 1 g/L and at pH 7.

S6. UV–Vis spectra

Figure S5 presents the UV–Vis spectra of PQ and TC solutions, used for adsorption isotherm analysis (initial concentrations of 5, 10, 25, 50, and 75 mg/L, with a composite dose of 1 g/L at pH 7). Solid lines represent the absorbance of the stock solutions, while dashed lines show the spectra after 24 hours of adsorption. The insets display the calibration curves (based on maximum absorbance at 260 nm for PQ and 356 nm for TC) used to calculate the residual concentrations.

**Figure S5.** UV–Vis spectra of (a) paraquat (PQ) and (b) tetracycline (TC) at initial concentrations ranging from 5 to 75 mg/L before (solid lines) and after 24 h of adsorption by PE–Ca–5%MOF (dashed lines) at a composite dose of 1 g/L and pH 7. Insets show the calibration curves constructed within their respective linear ranges from the maximum absorbance values at 260 nm for PQ and 356 nm for TC.

The limits of detection (LOD) and quantification (LOQ) for paraquat (PQ) and tetracycline (TC) by UV–Vis were determined from the linear calibration curves (see Table S5 for statistical parameters) using the standard deviation–to–slope method. Calibration data (n = 10) were fitted by least–squares regression ($A = SC + b$), and the standard deviation of the response (σ) was estimated as the residual standard deviation of the regression, calculated from the residual sum of squares (RSS) as $\sigma= \sqrt{RSS/(n-2)}$​. LOD and LOQ were then obtained as $LOD=3.3 \sigma/S$ and $LOQ = 10 \sigma/S$, respectively, and are reported in the same concentration units as the calibration. Using this approach, PQ showed a LOD and LOQ of 0.492 mg/L and 1.49 mg/L, respectively while TC showed a LOD and LOQ of 0.840 mg/L and 2.55 mg/L, respectively. Both analytes exhibited a linear UV–Vis response within the investigated calibration range, with PQ and TC showing linearity from the lowest calibration level up to 50 mg/L for PQ and 75 mg/L for TC, respectively. In Table S5 the “Intercept” corresponds to the predicted signal in the absence of analyte, while the “Slope” represents the method sensitivity, i.e., the change in signal per unit concentration; the reported ± values reflect the uncertainty of these fitted parameters. Finally, “LOD” (limit of detection) is the lowest concentration that can be reliably distinguished from the background, whereas “LOQ” (limit of quantification) is the lowest concentration that can be quantified with acceptable accuracy and precision, both reported in mg/L.

**Table S5.** Statistical parameters of the calibration curves used for quantification of paraquat (PQ) and tetracycline (TC).

| **Calibration Curves** | | | | | | | | |
| --- | --- | --- | --- | --- | --- | --- | --- | --- |
| **Pollutant** | **n** | **χ^2^** | **R^2^** | **Intercept** | **Slope** | **Residual sum of squares (RSS)** | **LOD**  **[mg/L]** | **LOQ**  **[mg/L]** |
| PQ | 10 | 0.99 | 0.99 | 0.01214 ± 0.0104 | 0.0677 ± 4.1×10^–4^ | 8.1×10^–4^ | 0.492 | 1.49 |
| TC | 10 | 0.99 | 0.99 | 0.00651 ± 0.00721 | 0.0343 ± 1.9×10^–4^ | 6.1×10^–4^ | 0.840 | 2.55 |

Figure S6 shows the comparison between the UV–Vis spectra of paraquat (PQ) and tetracycline (TC) solutions (10 mg/L, pH 7) after 24 h of contact with PE–Ca composites containing different MOF loadings and dispersion methods. For PQ (Figure S6a), both 1 wt% and 5 wt% water–dispersed composites significantly reduced the characteristic absorbance at 260 nm, with the PVP–assisted 1 wt% film reaching removal levels comparable to the 5 wt% water–dispersed film. The PVP–assisted foam exhibited the highest decrease in absorbance, indicating enhanced adsorption due to its increased porosity and improved MOF particle accessibility. For TC (Figure S6b), which absorbs maximally at 356 nm, adsorption was generally lower than for PQ due to its larger molecular size and reduced compatibility with the pectin network. Nevertheless, the PVP–assisted foam markedly improved removal compared to water–dispersed films, approaching the performance of the 5 wt% water–dispersed composite. These results highlight the importance of dispersion strategy and composite geometry in enhancing adsorption performance, especially for bulkier molecules such as TC.

**Figure S6.** UV–Vis spectra of (a) paraquat (PQ) and (b) tetracycline (TC) at an initial concentration of 10 mg/L after 24 h of adsorption (composite dose: 1 g/L, pH 7) by PE–Ca composites containing 1 wt% and 5 wt% Fe–BTC (water–dispersed) and 1 wt% Fe–BTC (PVP–assisted) in film and foam geometries.

References

1. Vareda, J.P. On Validity, Physical Meaning, Mechanism Insights and Regression of Adsorption Kinetic Models. *J Mol Liq* 2023, *376*, 121416, doi:10.1016/j.molliq.2023.121416.

2. Cavanaugh, J.E.; Neath, A.A. The Akaike Information Criterion: Background, Derivation, Properties, Application, Interpretation, and Refinements. *Wiley Interdiscip Rev Comput Stat* 2019, *11*.

3. Neath, A.A.; Cavanaugh, J.E. The Bayesian Information Criterion: Background, Derivation, and Applications. *Wiley Interdiscip Rev Comput Stat* 2012, *4*, 199–203, doi:10.1002/wics.199.

4. Synytsya, A.; Čopíková, J.; Matějka, P.; Machovič, V. Fourier Transform Raman and Infrared Spectroscopy of Pectins. *Carbohydr Polym* 2003, *54*, 97–106, doi:10.1016/S0144–8617(03)00158–9.

5. Coin, F.; Larrañaga, A.; Cerveny, S. Structural Characterization of Low Methoxyl Pectin–Based Adsorbents: The Role of Water on Pectin Structure. *Carbohydrate Polymer Technologies and Applications* 2025, *11*, doi:10.1016/j.carpta.2025.100885.

6. Gohil, R.M. Synergistic Blends of Natural Polymers, Pectin and Sodium Alginate. *J Appl Polym Sci* 2011, *120*, 2324–2336, doi:10.1002/app.33422.

7. Chen, G.; Leng, X.; Luo, J.; You, L.; Qu, C.; Dong, X.; Huang, H.; Yin, X.; Ni, J. In Vitro Toxicity Study of a Porous Iron(III) Metal–Organic Framework. *Molecules* 2019, *24*, doi:10.3390/molecules24071211.

**Disclaimer/Publisher’s Note:** The statements, opinions and data contained in all publications are solely those of the individual author(s) and contributor(s) and not of MDPI and/or the editor(s). MDPI and/or the editor(s) disclaim responsibility for any injury to people or property resulting from any ideas, methods, instructions or products referred to in the content.
